# A Description of Acute Renal Failure and Nephrolithiasis Associated With Sodium–Glucose Co-Transporter 2 Inhibitor Use: A VigiBase Study

**DOI:** 10.3389/fphar.2022.925805

**Published:** 2022-08-08

**Authors:** Ioana Frent, Daniel Leucuta, Camelia Bucsa, Andreea Farcas, Florin Casoinic, Cristina Mogosan

**Affiliations:** ^1^ Department of Pharmacology, Physiology and Physiopathology, Faculty of Pharmacy, “Iuliu Haţieganu” University of Medicine and Pharmacy, Cluj-Napoca, Romania; ^2^ Department of Medical Informatics and Biostatistics, “Iuliu Hatieganu” University of Medicine and Pharmacy, Cluj-Napoca, Romania; ^3^ Pharmacovigilance Research Center, “Iuliu Hatieganu” University of Medicine and Pharmacy, Cluj-Napoca, Romania; ^4^ Department of Internal Medicine, “Iuliu Hațieganu” University of Medicine and Pharmacy, Cluj-Napoca, Romania

**Keywords:** SGLT2I, acute kidney injury, acute renal failure, nephrolithiasis, drug-induced acute kidney injury, drug-induced nephrolithiasis, disproportionality analysis, VigiBase

## Abstract

**Background:** The Food and Drug Administration issued a warning on the risk of acute kidney injury and a signal of nephrolithiasis for patients using sodium–glucose co-transporter 2 inhibitors (SGLT2i). We performed a descriptive analysis on acute renal failure (ARF) and nephrolithiasis cases reported to SGLT2i in the VigiBase^®^, in the scope of characterizing the patients and reactions and to report on the disproportionality analysis.

**Methods:** We analyzed all ARF and nephrolithiasis reports for SGLT2i in VigiBase from inception to September 2021. ARF cases were defined as reports containing at least one of the preferred terms (PTs) included in the ARF narrow Medical Dictionary for Regulatory Activities Standardised Queries (MedDRA SMQ). SGLT2i exposure was considered for reports with at least one gliflozin as a suspected/interacting drug. We characterized the patients, reporters, and reactions, and we present the proportional reporting ratio (PRR).

**Results:** Of 27,370,413 total reports in VigiBase, we found 3,972 ARF reactions to gliflozins as suspected/interacting drugs in 3,751 patients and 231 nephrolithiasis reactions in 227 patients. Most cases were reported from American regions (3057; 81.49%), for patients of age group 45–64 years (1590; 59%). About 30% (1156) of the ARF reports were registered in 2018, most from spontaneous reporting, and from consumers followed by healthcare professionals (2,235; 61% and 1440; 38%, respectively). Canagliflozin was the most involved gliflozin in the ARF and nephrolithiasis cases (2,640; 67% and 109; 47%, respectively). The great majority of ARF and nephrolithiasis reports were serious (3,761; 95% and 182; 79%, respectively). Of the total ARF cases reported, 51 had fatal outcome, while 152 had not recovered/not resolved outcome. No fatal outcome was reported for nephrolithiasis. Disproportionality analysis in full database showed a PRR of 4.68 (95% CI 4.53–4.83) for all gliflozins–ARF and a PRR of 3.44 (95% CI 3.00–3.95) for all gliflozins–nephrolithiasis.

**Conclusion:** Most of ARF reports associated with gliflozins were serious, with an important number of cases with fatal outcome. A drug safety signal was found between ARF narrow SMQ and gliflozins. Also, gliflozins were associated with an increase in the proportion of nephrolithiasis reports compared to other medications.

## 1 Introduction

The benefit–risk balance of sodium–glucose co-transporter 2 inhibitors (SGLT2i, gliflozins), a relatively new glucose-lowering agent (GLA) class, was extensively studied over the last years. SGLT2i have proven to have beneficial effects beyond glycemic control, on metabolic, cardiovascular, and renal outcomes (Minze et al., 2018). Three cardiovascular clinical trials, (EMPA-REG, CANVAS, and DECLARE-TIMI 58 trial), reported beneficial renal outcomes (AY et al., 2018). Two kidney clinical trials (CREDENCE and DAPA_CKD) demonstrated that SGLT2 inhibitors can reduce the risk of worsening chronic kidney disease (CKD) (Giorgino et al., 2020). In contrast to this renoprotective benefit proven during clinical trials, several case reports on acute kidney injury (AKI) associated with SGLT2i were reported and triggered a strengthening of the warning on the risk of AKI issued by the Food and Drug Administration (FDA) (U.S. Food and Drug Administration, 2015; U.S. Food and Drug Administration, 2016). In addition, nephrolithiasis is another possible adverse drug reaction (ADR) of SGLT2i at the renal level. FDA also issued a warning on this potential risk, raising concerns regarding renal safety of these medications (U.S. Food and Drug Administration, 2017).

AKI is defined by an abrupt decrease in kidney function with important clinical consequences, including increased risk of death. AKI includes, but is not limited to, acute renal failure (ARF) and other, less severe conditions (Kellum et al., 2012). As a syndrome, AKI includes patients without actual damage to the kidney but with functional impairment (Kellum et al., 2012). Patients with diabetes are known to have a higher susceptibility to AKI/ARF (Wang et al., 2021), but also medications are a common cause of AKI/ARF, especially for patients admitted to hospital wards and the intensive care unit (Perazella and Rosner, 2022a).

The risk of developing renal impairment with SGLT2i and the role of plausible mechanisms have to be established yet (Szalat et al., 2018). SGLT2i may induce excessive diuresis which can lead to intravascular volume depletion, particularly in hemodynamically unstable and volume-depleted patients (Perlman et al., 2017; Szalat et al., 2018). Reduced trans-glomerular pressure with a modest decline in kidney function, a characteristic of SGLT2i, is on the long-term renal protective. The acute decrease in estimated glomerular filtration rate (eGFR) was attributed to the effect of proximal tubular natriuresis on tubuloglomerular feedback, leading to reversible intrarenal hemodynamic effects, including afferent arteriole vasoconstriction (Cherney et al., 2014; Sridhar et al., 2020; Meraz-Muñoz et al., 2021). Lastly, SLGT2i increase medullary oxygen consumption, increasing the risk for hypoxia (Perlman et al., 2017), especially with concomitant use of agents impairing medullary oxygenation, such as nonsteroidal antiinflammatory drugs (NSAIDs) and radiocontrast agents (Szalat et al., 2018).

The clinical implications of the acute decrease in eGFR were unknown and led to concerns about the safety of SGLT2i because observational reports suggested an increase in the risk of AKI (Perlman et al., 2017). On the other hand, a retrospective cohort study found that there were no differences observed in the incident AKI in SGLT2i versus other GLAs (Rampersad et al., 2020). A systematic review and meta-analysis showed that SGLT2i even reduced the odds of suffering AKI with and without hospitalization in randomized trials and in the real-world setting, despite the fact that more AEs related to hypovolemia are reported (Menne et al., 2019). In addition, some propensity score analysis comparing SGLT2i with other antidiabetics (dipeptidyl peptidase 4 inhibitors) reported that AKI risk was reduced in SGLT2i users, but the mechanism is unknown yet (Nadkarni et al., 2017; Sridhar et al., 2020).

In addition, SGLT2i treatment could lower the risk for incident and recurrent kidney stones in people with type 2 diabetes, as recent evidence suggests (Kristensen et al., 2021). SGLT2i induce osmotic diuresis, increased urinary flow, polyuria, and urine dilution that may even reduce the risk of nephrolithiasis (Kristensen et al., 2021). However, a potential signal of nephrolithiasis was detected earlier by the FDA in patients using SGLT2 inhibitors (U.S. Food and Drug Administration, 2017). It was also suggested that SGLT2i can cause hyperuricosuria (Chino et al., 2014; Novikov et al., 2019), which may confer a greater risk for specific types of kidney stones. Diabetes mellitus is also a known risk factor for nephrolithiasis (Aune et al., 2018).

Despite the fact that the risk of AKI and nephrolithiasis was not confirmed by observational database studies of large cohorts, reports of AKI and nephrolithiasis associated with SGLT2i use continue to be submitted to VigiBase (2487 and 231 reactions, respectively, by September 2021), the World Health Organization (WHO) unique global database of individual case safety reports (ICSRs). VigiBase includes reports from around 140 countries representing over 90% of the world’s population and is maintained by the Uppsala Monitoring Centre (Uppsala, Sweden). The database contained more than 27 million ICSRs that has been submitted by national pharmacovigilance centers since 1967. Although VigiBase data cannot offer evidence on the causality relationship between the gliflozins and the events, analysis on the disproportionality of events reported for a particular drug versus the rest of the database and versus a restrictive diabetic therapeutic area can still be informative and add value to better characterize the safety profile of this class of drugs. Also, specific information on patients and reactions reported may help in contouring the common ground of the reactions reported. The objective of our study was to perform such analyses in the VigiBase^®^, on ARF and nephrolithiasis reported to SGLT2i versus all ADRs and ADRs of all antidiabetics, in the scope of characterizing the patients and reactions and to investigate their relationship through disproportionality analysis in the largest international pharmacovigilance database.

## 2 Methods

### 2.1 Study Population and Design

This is an observational study that characterizes the reported ARF and nephrolithiasis reactions related to gliflozins use and presents their disproportionality analysis when compared to all ADRs and to ADRs of all antidiabetics (ATC A10) in the VigiBase from inception to 31 August 2021.

The adverse events captured in the ICSRs have been coded according to the latest version of the Medical Dictionary for Regulatory Activities (MedDRA) at the time of reporting.

### 2.2 Information

Following variables on the reports were received from WHO: region of origin, date of report, reporter qualification, report source, serious, seriousness criteria per reaction, patient’s characteristics (sex and age group), drugs (indication, start and end dates, dosage, and route of administration), and reactions reported (MedDRA terms, onset date and end date, time to onset, reaction outcome, and dechallenge/rechallenge action). One ICSR can include more than one adverse reaction and more than one suspected or interacting drugs.

### 2.3 Data Management

All ARF and nephrolithiasis reactions with canagliflozin, dapagliflozin, empagliflozin, ertugliflozin, ipragliflozin, and/or luseogliflozin as suspected or interacting drugs until 31 August 2021 were received from VigiBase. In addition, the disproportionality analysis of each preferred term (PT, the term used to describe an adverse event in MedDRA) associated with each of the gliflozins as compared to all ADR reports and compared to all antidiabetics reports were received from VigiBase. We hypothesized that it is possible that some cases could be reported with a PT different from AKI or ARF, and decided to use MedDRA Standardised Medical Queries (SMQs), which are validated, predetermined sets of MedDRA terms grouped together to support safety analysis.^1^ We identified the acute renal failure SMQ broad and narrow and we decided to use the narrow search as this is more specific (cases highly likely to be related to a specific condition). Narrow search on SMQ “Acute Renal Failure” yielded 19 PTs as follows: Acute kidney injury, Acute phosphate nephropathy, Anuria, Azothemia, Continuous hemodiafiltration, Dialysis, Fetal renal impairment, Hemodialysis, Hemofiltration, Neonatal anuria, Nephropathy toxic, Oliguria, Peritoneal dialysis, Prerenal failure, Renal failure, Renal failure neonatal, Renal impairment, Renal impairment neonatal, and Subacute kidney injury. All these PTs were included in the analysis.

The reports of ARF with fatal outcome were further analyzed with the aim of identifying and characterizing co-suspect/concomitant medications, concurrent conditions and potential risk factors for fatal ARF.

Regarding nephrolithiasis, we used only the PT with the same name.

Exposure to gliflozins was defined as the mention in the report of at least one of the following: canagliflozin, dapagliflozin, empagliflozin, ertugliflozin, ipragliflozin, and/or luseogliflozin as suspect or interacting medication.

### 2.4 Data Analysis

Descriptive statistics were used to summarize the baseline characteristics of the reports, patients, drugs, and reactions for the ICSRs containing adverse events from the ARF SMQ narrow (ARF reactions hereafter) and nephrolithiasis reactions. The comorbidities of the patients were retrieved from the field of indication for all drugs included in the analyzed ICSRs.

The disproportionality of selected reactions associated with the use of the six gliflozins was studied in a case–non-case analysis individually for each PT. The proportion of ARF/nephrolithiasis reactions reported for gliflozins was compared with the proportion of the ARF/nephrolithiasis reactions reported for all other drugs in VigiBase. Additionally, as diabetes mellitus itself is a known risk factor for development of kidney disease, a sensitivity analysis was performed and the database was restricted to the diabetic therapeutic area (i.e., considering only medicines used in diabetes) as the background, to take into account the increased baseline risk of ARF and nephrolithiasis. Furthermore, we excluded from the database all reactions with co-suspected drugs to limit the innocent bystander effect with gliflozins.

The proportional reporting ratio (PRR) was used as a measure of disproportionate reporting. The data were provided by the Uppsala Monitoring Centre for individual gliflozins and PTs. We calculated the PRR for the gliflozin class and ARF reactions and nephrolithiasis as described in the Supplementary Tables 1, 2 on the data provided by the WHO Uppsala Monitoring Center(Proportional Reporting Ratio, 2022).

A PRR value ≥ 1 associated with ≥5 cases was considered a positive association between the reaction and gliflozins (Slattery et al., 2013).

Microsoft Excel was used to compute the PRR values that were not provided by WHO Uppsala Monitoring Center and to tabulate relevant data.

## 3 Results

### 3.1 Case Selection

**FIGURE 1:**
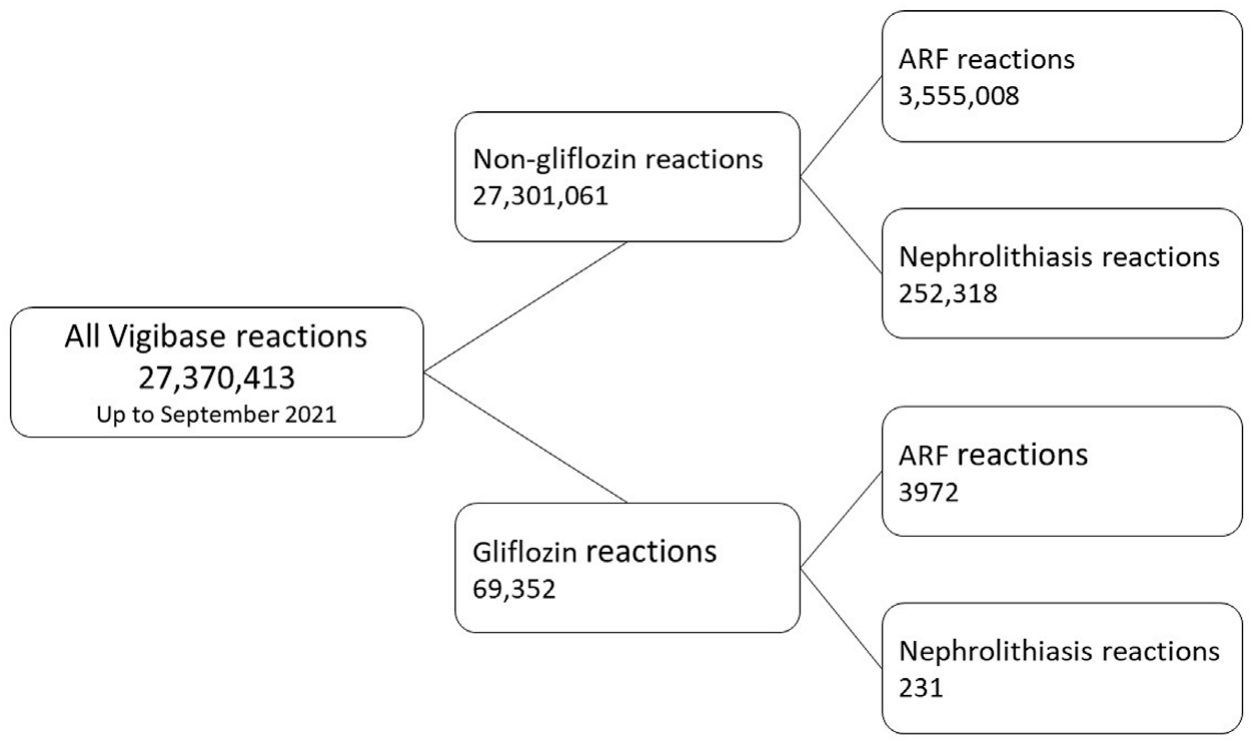
Flowchart of the ICSRs included in the analysis.

A flowchart of the study is presented in Figure 1. VigiBase contained 27,370,413 reports until 31 August 2021. Exposure to gliflozins as suspected/interacting drug was found in 69,352 reports.

### 3.2 Reports and ADR Characteristics

We identified 3972 ARF reports to gliflozins as suspected/interacting drugs in 3751 patients and 231 nephrolithiasis reactions in 227 patients (more than one gliflozin may be suspected for one reaction and more than one PT may be reported in case of ARF SMQ).

The characteristics of reports, patients, and reactions are shown in Table 1. The most cases with available information were reported in the regions of America (3057; 81.49%), in the category of 45–64 years of age (1590; 59%) and male gender was more frequent (1852; 57% and 114; 56% in ARF and nephrolithiasis reports, respectively).

**TABLE 1:** Characteristics of ARF and nephrolithiasis cases/reactions.

Characteristic	ARF n (%)	Nephrolithiasis n (%)
Total reports (ICSRs)	3.751	227
Total number of reactions	3.972	231
**Patients**		
**Age group**		
0–27 days	1 (0.03%)	0
28 days–23 months	1 (0.03%)	0
2–18 years	0 (0%)	0 (0%)
18–44 years	344 (12.69%)	9 (7.08%)
45–64 years	1590 (58.67%)	76 (59.84%)
65–74 years	548 (20.24%)	32 (25.19%)
>75 years	226 (8.33%)	10 (7.87%)
Unknown	1041	100
**Gender**		
Female	1397 (42.99%)	90 (44.11%)
Male	1852 (56.94%)	114 (55.88%)
Unknown	502	4
**Reports**		
**Reporting year**		
2011	1 (0.02%)	0
2012	1 (0.02%)	1 (0.44%)
2013	8 (0.21%)	0
2014	67 (1.78%)	10 (4.40%)
2015	341 (9.09%)	53 (23.34%)
2016	372 (9.91%)	32 (14.09%)
2017	681 (18.15%)	36 (15.85%)
2018	1156 (30.81%)	34 (14.95%)
2019	737 (19.64%)	27 (11.89%)
2020	218 (5.81%)	20 (8.81%)
2021	169 (4.5%)	14 (6.16%)
**WHO region of report**		
Europe	448 (11.94%)	24 (11%)
Eastern Mediterranean region	2 (0.05%)	1 (0.45%)
America	3057 (81.49%)	184 (84.4%)
Western Pacific	229 (6.1%)	9 (4.12%)
South-East Asia	9 (0.23%)	0
Africa	6 (0.15%)	0
*Report source (N)	3755	227
Spontaneous	3605 (96.08%)	206 (90.74%)
Study	139 (3.7%)	21 (9.25%)
Other	4 (0.10%)	0
PMS	2 (0.05%)	0
Other	2 (0.05%)	0
Unknown	3	0
*Notifier type (N)	3827	258
Physician	996 (26.04%)	68 (58.62%)
Pharmacist	179 (4.86%)	2 (1.72%)
Other health professional	265 (7.19%)	14 (12.05%)
Consumer/non-health professional	2235 (60.86%)	32 (27.58%)
Lawyer	8 (2.17%)	0
Unknown	144	142
**Drugs**		
**Canagliflozin**	**2640 (66.81%)**	**109 (47.18%)**
10 mg	1	0
50 mg	2	0
90 mg	1	0
100 mg	514	29
150 mg	6	0
200 mg	12	1
300 mg	567	18
Unknown	1412	55
Canagliflozin + metformin	121	6
Canagliflozin + teneligliptin	4	0
**Dapagliflozin**	622 (15.74%)	55 (23.80%)
5 mg	87	12
10 mg	205	17
15 mg	1	0
20 mg	1	1
25 mg	1	-
Unknown	238	18
Dapagliflozin + metformin	88	7
Dapagliflozin + saxagliptin	1	0
**Empagliflozin**	606 (15.33%)	65 (28.13%)
5 mg	0	0
10 mg	179	19
12.5 mg	17	1
20 mg	1	0
25 mg	114	9
50 mg	1	0
Unknown	239	29
Empagliflozin + metformin	36	2
Empagliflozin + linagliptin	18	5
Empagliflozin + linagliptin + metformin	1	0
**Ertugliflozin**	15 (0.37%)	1 (0.43%)
Ertugliflozin	11	0
Ertugliflozin + metformin	2	0
Ertugliflozin + sitagliptin	2	1
**Ipragliflozin**	52 (1.26%)	0
25 mg	2	0
50 mg	42	0
75 mg	1	0
100 mg	1	0
Unknown	6	0
**Luseogliflozin**	16 (0.40%)	1 (0.43%)
2.5 mg	14	1
5 mg	2	0
**Events**	3972	231
**ARF PT**	n (%)	
Acute kidney injury	2487 (62.61%)	
Acute phosphate nephropathy	0	
Anuria	16 (0.40%)	
Azotemia	6 (0.15%)	
Continuous hemodiafiltration	0	
Dialysis	23 (0.57%)	
Fetal renal impairment	0	
Hemodialysis	5 (0.12%)	
Hemofiltration	1 (0.02%)	
Neonatal anuria	0	
Nephropathy toxic	2 (0.05%)	
Oliguria	8 (0.20%)	
Peritoneal dialysis	1 (0.02%)	
Prerenal failure	15 (0.37%)	
Renal failure	907 (22.83%)	
Renal failure neonatal	0	
Renal impairment	501(12.61%)	
Renal impairment neonatal	0	
Subacute kidney injury	0	
**Serious**		
Serious	3761(95.79%)	182 (79.13%)
Non-serious	165 (4.2%)	48(20.86%)
Unknown	46	1
**Seriousness**		
Death	134 (3.7%)	1 (0.55%)
Life threatening	344 (9.4%)	6 (3.35%)
Caused/prolonged hospitalization	2102 (57.7%)	79 (44.13%)
Disabling/incapacitating	8 (0.2%)	3 (1.67%)
Other	1054 (28.9%)	90 (50.27%)
Unknown	330	52
**Time to onset**		
<1 week	59 (18.32%)	5 (21.73%)
1–2 weeks	25 (7.76%)	0
0.5–1 month	33 (10.24%)	1 (4.34%)
1–2 months	47 (14.59%)	3 (13.04%)
2–3 months	36 (11.18%)	3(13.04%)
3–6 months	43 (13.35%)	0
6–12 months	33 (10.24%)	6 (26.08%)
12–24 months	32 (9.93%)	3(13.04%)
>24 months	14 (4.34%)	2(8,69%)
Unknown	280	204
**Reaction (event) duration (total known n)**	141	8
<1 week	64 (45.39%)	3(37.5%)
1–2 weeks	32(22.69%)	0
0.5–1 month	19(13.47%)	0
1–2 months	12(8.51%)	3(37.5%)
2–3 months	5(3.54%)	0
3–6 months	5(3.54%)	0
6–12 months	10.70)	2(25%)
12–24 months	3(2.12%)	0
Unknown	3827	219
**Outcome (total known n)**	**n =1882**	**n = 80**
Fatal	51 (2.70%)	0
Not recovered/not resolved	152 (8.07%)	31 (38.75%)
Recovered/resolved with sequelae	19 (1%)	0
Recovering/resolving	801 (42.56%)	9 (11.25%)
Recovered/resolved	859 (45.64%)	40 (50%)
Unknown + data not available	1865	146
**De-challenge performed, n (%)**		
Dose reduced	13 (0.64%)	1 (0.73%)
Drug withdrawn	1843 (91.32%)	85 (62.5%)
Drug increased	6 (2.99%)	2 (1.47%)
Drug not changed	143 (7.13%)	48 (35.29%)
Unknown	1579	62
Not applicable	92	0
**De-challenge outcome (Total known n)**	**n =1865**	**n = 79**
Reaction abated	1724 (92.43%)	46 (58.22%)
No effect observed	141 (7.56%)	33 (41.77%)
Not applicable	10	1
Effect unknown	1740	117
**Rechallenge action**		
No rechallenge	1	0
Rechallenge	2793	116
**Rechallenge outcome**		
Reaction recurred	3	0
No recurrence	942	42
Effect unknown	1848	74

ARF, acute renal failure; PT, preferred term; WHO, World Health Organization; N, total number of cases; n, number of cases in a given category; % is calculated of N or of the total known number in a given category; ICSR, individual case safety report; AE, adverse event; *, one AE may have one or more reporters; one ICSR may include more than one AE reported; one ICSR may have as suspect or interacting drugs more than one gliflozins—this led to a higher number of notifiers and report types than the total number of ICSRs; de-challenge, reports where dose of gliflozin was decreased/drug withdrawn; if two or more values of time-to-onset were reported in one ICSR, the most decreased value was chosen; serious, seriousness counted per reaction; outcome, the worse reported outcome in a chosen report.

Most of the ARF reports came from spontaneous reporting, the majority from consumers followed by healthcare professionals (2235; 60.86% and 1.440; 38.09% from available data, respectively with 59.57% coming from consumers only). The first ARF reports were registered in 2011 and their number increased until 2018 (1156, around 31% of the total reports) and then declined until 2021 (169 reports by September) (Table 1). For nephrolithiasis, the highest number of reports were registered in 2015 (53 cases) and slightly decreased in the years after.

For the ARF analysis, we found that canagliflozin was the most frequently involved gliflozin (2640 cases; 66.81%) followed by dapagliflozin (622 cases; 15.74%) and empagliflozin (606 cases; 15.33%). Canagliflozin was the most involved gliflozin in the nephrolithiasis cases as well (109 cases; 47.18%), followed by empagliflozin (65 cases; 28.13%) and dapagliflozin (55 cases; 23.80%) (Table 1).

**FIGURE 2:**
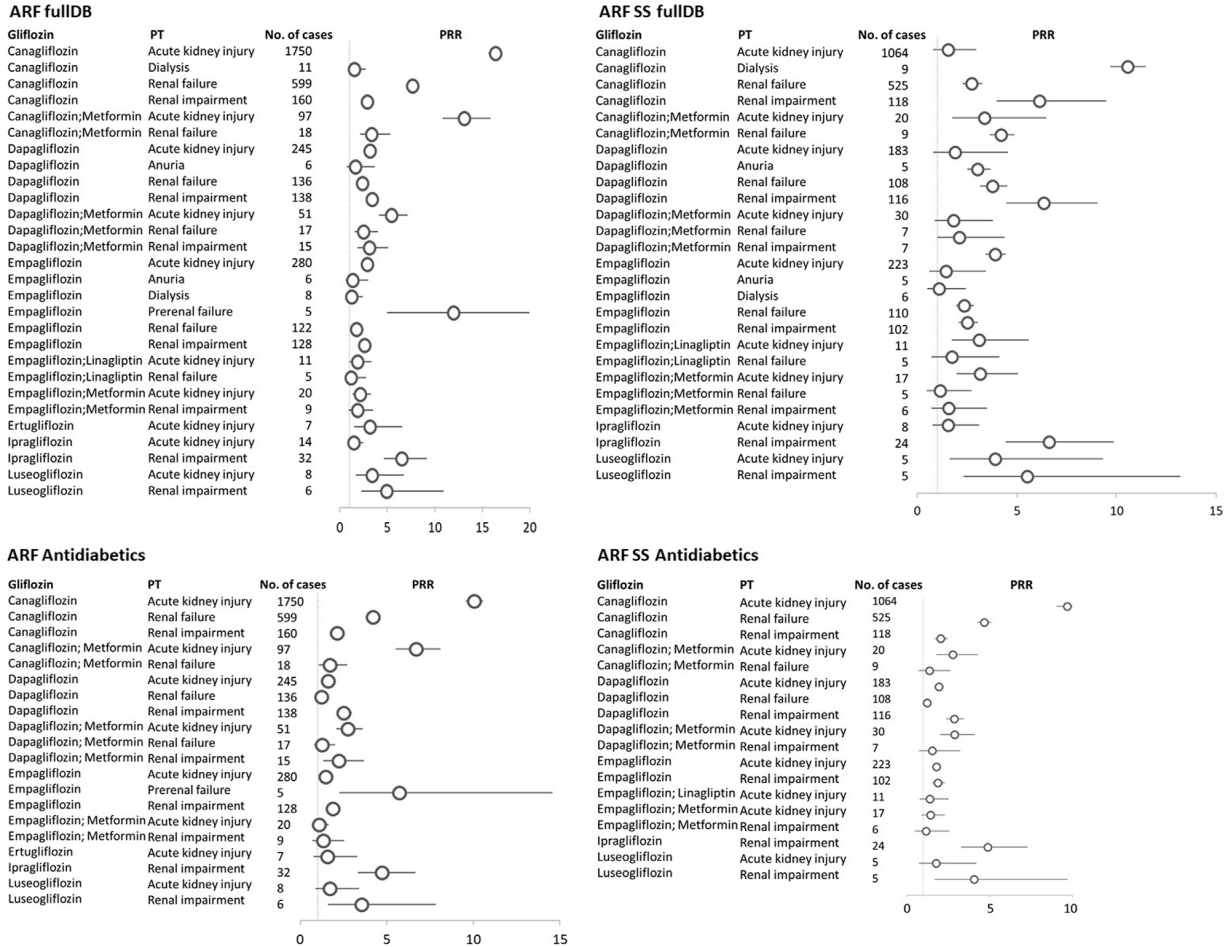
Gliflozins-ARF narrow SMQ reactions with the proportional reporting ratio (PRR) greater than 1 and minimum 5 reactions were reported. ARF fullDB, acute renal failure narrow SMQ reactions with PRR calculated against all reports in VigiBase; ARF SS fullDB, acute renal failure narrow SMQ reactions with PRR calculated against all reports in VigiBase where gliflozins are the only suspect in the respective reaction; ARF Antidiabetics, acute renal failure narrow SMQ reactions with PRR calculated against all reports to antidiabetics in VigiBase; ARF SS Antidiabetics, acute renal failure narrow SMQ reactions with PRR calculated against all reports to antidiabetics in VigiBase where gliflozins are the only suspect in the respective reaction; PT, MedDRA preferred term; circles represent PRR, and horizontal lines represent 95% confidence interval.

Of the 19 PTs included in the ARF SMQ narrow, we found 12 PTs reported for gliflozins. AKI (2387: 62.61%), renal failure (907; 22.83%), and renal impairment (501; 12.61%) were the most reported PTs for each gliflozin. Most of the reports were with canagliflozin (1750 AKI, 599 renal failure and 160 renal impairment), followed by empagliflozin (280 AKI, 128 renal impairment and 122 renal failure) and dapagliflozin (245 AKI, 138 renal impairment and 136 renal failure), (Table 1; Figure 2).

Information on time-to-onset of the reaction was available in 322 (8%) cases for ARF and in 158 reports ARF occurred after the first 8-week period of starting therapy (49% of the cases with this information available). In 45% of the 141 cases with available information, the reaction duration was less than 1 week for ARF. In 23 cases with information available for nephrolithiasis, we found the most cases reported within 6–12 months from treatment start (6 cases) followed by the interval within first week (5 cases) (Table 1).

The great majority of ARF reports were serious (95.79%) with more than half (57.7%) causing/prolonging hospitalization. Of the total ARF reported, 51 had fatal outcome (2.7% from available data), while 152 (8.07%) had not recovered/not resolved outcome. However, the vast majority of the cases have a favorable outcome, recovered or recovering at the moment of reporting (88.2% of known data). In 1724 of the ARF reactions, reaction abated after gliflozin withdrawal (positive de-challenge in 43.40% of the total ARF reactions) and also 3 cases with positive re-challenge were reported (Table 1).

The most frequent co-suspect and interacting drugs reported in the 3751 ARF ICSRs were: other antidiabetics (biguanide, insulin, DPP-4 inhibitors, GLP-1 receptor agonists, and sulphonylurea), diuretics and antihypertensive drugs. We found an important number of patients using diuretics (n = 125), ACE inhibitors and ARB blockers (n = 163), and NSAIDs (n = 43) who had ARF (Table 2) The most frequent comorbidities of the patients with ARF were hypertension, cardiac disorders, depression and anxiety, hyperlipidemia, and pain. Associated ketoacidosis was frequently reported (27.87% of reports with ARF reactions), followed by different types of infections. If we cumulate the number of infections, these become the most frequently reported PTs (30.84% of reports with ARF reactions) (Table 2) Regarding associated conditions with ARF that induce plasmatic volume depletion, we found dehydration, diarrhea, and vomiting (n = 437 associated PTs), cardiac failure (n = 86 associated diseases), hypovolemia (n = 25 associated PTs), or concomitant use of diuretics (n = 124).

**TABLE 2:** Concomitant medication (co-suspect and interacting) and associated conditions in ARF and nephrolithiasis reports.

	ARF N	Nephrolithiasis N
**Concomitant medication**		
Biguanide	690	10
Insulin	209	0
DPP-4 inhibitors	134	0
*Diuretics	125	0
*ACE inhibitors	110	0
GLP-1 receptor agonist	60	3
*ARB	53	0
Sulfonylurea	47	0
*NSAIDS	43	0
Statin	15	0
Acetylsalicylic acid	9	0
**Allopurinol	2	1
**Associated diseases**	**N**	**N**
** **Hypertension	379	20
** **Blood cholesterol increased	173	5
** **Pain	164	12
** **Cardiac disorder	119	2
** **Cardiac failure	86	1
** **Thyroid disorder	77	1
** **Depression	71	4
** **Infection	45	0
** **Anxiety	42	2
** **Gastroesophageal reflux disease	42	6
** **Renal disorders and chronic kidney disease	20	2
** **Urinary tract infection	16	0
** **Blood pressure decreased	16	0
** **Fluid retention	15	2
** **Diarrhea	4	1
** **Vomiting	4	0
** **Gout	16	3
** **Vitamin D deficiency	11	3
** **Obesity	4	2
** **Prostatic hypertrophy	12	1
** **Colitis ulcerative	2	1
** **Sepsis	2	0
**Associated reported PTs**	**N**	**N**
** **Diabetic ketoacidosis, ketoacidosis, euglycemic diabetic ketoacidosis	1107	15
** **Infection (including UTI)	428	60
** **Dehydration, vomiting, and diarrhea	437	11
** **Sepsis, urosepsis, *Escherichia* sepsis, staphylococcal sepsis, bacterial sepsis, streptococcal sepsis, wound sepsis, pulmonary sepsis, and *Candida* sepsis	308	18
** **Osteomyelitis and acute osteomyelitis	214	0
** **Metabolic acidosis and lactic acidosis	228	2
** **Urinary tract infection, kidney infection, and pyelonephritis	275	45
** **Toe/leg/foot/limb amputation and amputation	319	1
** **Cellulitis	89	0
** **Gangrene	81	0
** **Encephalopathy and metabolic Encephalopathy	71	0
** **Hyperkalemia	69	1
** **Septic shock	66	1
** **Myocardial infarction	61	1
** **Fournier’s gangrene	45	0
** **Hypotension	45	1
** **Pancreatitis acute	28	0
** **Hypovolemia	25	0
** **Cerebrovascular accident	24	0
** **Urinary tract obstruction	5	2

ARF, acute renal failure; PTs, preferred terms; ICSR, individual case safety report; N, total number of cases; n, number of cases in a given category; DPP-4, dipeptidyl peptidase-4; GLP-1, glucagon-like peptide-1; ACE, angiotensin-converting enzyme; ARB, angiotensin receptor blocker; NSAID, nonsteroidal antiinflammatory drugs; UTI, urinary tract infection; *, drugs that may induce acute kidney injury (Perazella and Rosner, 2022b); **, drugs that may induce nephrolithiasis (Daudon et al., 2018); drug indication was not mentioned in all reports. One ICSR may include more than one ARF PT reported, and one ICSR may have as suspect or interacting drugs more than one gliflozins, and this may lead to a discrepancy between the number of cases and number of drugs.

For nephrolithiasis, the majority of reports were also serious (79%) and 50% of the cases with this information available had a favorable outcome. No fatal cases were reported. We found 46 nephrolithiasis reactions with positive de-challenge (58.22% of the available data), but no cases with positive re-challenge were reported (Table 1). Urinary/renal infections were the most frequent associated adverse event with nephrolithiasis (52 reports, 22.51%).

No dose–response pattern was observed for ARF and nephrolithiasis (Table 1).

### 3.3 Fatal Outcome Analysis

Of the total of 3972 ARF ICSRs, 134 (3.7%) deaths were reported as seriousness criteria and a fatal outcome due to ARF reaction was reported for 51 patients (1.35%).

The characteristics of reports, patients, and reactions are shown in Table 3. Most of the reports came from spontaneous reporting and half from healthcare professionals. Therefore, half of the fatal ARF cases were considered medically confirmed.

**TABLE 3:** Characteristics of fatal ARF.

Characteristic	ARF n (%)
Total reports (ICSRs)	51
Total number of reactions	54
**Patients**	
**Age group**	
18–44	2 (4.54%)
45–64	15 (34.09%)
65–74	15 (34.09%)
>75	12 (27.27%)
Unknown	7
**Gender**	
Female	20 (40.81%)
Male	29 (59.18%)
Unknown	2
**Reports**	
Reporting year	
2011	0
2012	0
2013	0
2014	1 (1.96%)
2015	7 (13.72%)
2016	5 (9.80%)
2017	5 (9.80%)
2018	21 (41.17%)
2019	4 (7.84%)
2020	4 (7.84%)
2021	4 (7.84%)
**WHO region of report**	
Europe	11 (21.56%)
America	32 (62.74%)
Western Pacific	7 (13.72%)
Africa	1 (1.96%)
**Report source (N)**	
Spontaneous	48 (94.11%)
Study	3 (5.88%)
*Notifier type (N)	
Physician	22 (43.13%)
Other health professional	4 (7.84%)
Consumer/non-health professional	25 (49.01%)
**Drugs**	
Canagliflozin	26 (50.98%)
100 mg	10 (62.5%)
300 mg	6 (37.5%)
Dapagliflozin	16 (31.37%)
5 mg	3 (23.07%)
10 mg	10 (76.92%)
Dapagliflozin + metformin	4 (7.84%)
Empagliflozin	9 (17.64%)
10 mg	4 (57.14%)
25 mg	3 (42.85%)
Empagliflozin + metformin	1 (1.96%)
**Events**	
**ARF PT**	54
Acute kidney injury	30 (55.55%)
Dialysis	1 (1.85%)
Hemofiltration	1 (1.85%)
Renal failure	20 (37.03%)
Renal impairment	2 (3.73%)
Acute phosphate nephropathy, anuria, azotemia, continuous hemodiafiltration, fetal renal impairment, hemodialysis, neonatal anuria, nephropathy toxic, oliguria, peritoneal dialysis, prerenal failure, renal failure neonatal, renal impairment neonatal, and subacute kidney injury	0
**Time to onset**	
<1 week	3 (30%)
1–2 weeks	0
0.5–1 month	2 (20%)
1–2 months	0
2–3 months	2 (20%)
3–6 months	1 (10%)
6–12 months	0
12–24 months	1 (10%)
>24 months	1 (10%)
**De-challenge performed, n**	—
Drug withdrawn	13 (86.66%)
Drug not changed	2 (13.33%)

ARF, acute renal failure; PT, preferred term; AE, adverse event; ICSR, individual case safety report; WHO, World Health Organization; N, total number of cases; n, number of cases in a given category; % is calculated of N or of the total known number in a given category; *, one AE may have one or more reporters; one ICSR may include more than one AE reported; one ICSR may have as suspect or interacting drugs more than one gliflozins. This led to a higher number of notifiers than the total number of ICSRs; de-challenge, reports where dose of gliflozin was decreased/drug withdrawn; if two or more values of time to onset were reported in one ICSR, the most decreased value was chosen; serious, seriousness counted per reaction; outcome, the worse reported outcome in a chosen report.

In 32 fatal ARF reports (62.74%), the only suspect drug was one of the gliflozins. Most of the ARF cases were reported as AKI (n = 30) followed by renal failure (n = 20), renal impairment (n = 2), hemofiltration (n = 1), and dialysis (n = 1). In 16 (31%) cases, the only fatal reaction reported was as an ARF PT: AKI (n = 9), renal failure (n = 6), or renal impairment (n = 1).

In the majority of the reports (n = 35, 68%), other associated conditions were also considered responsible for this fatal outcome along with ARF. The most frequent associated causes of death were metabolic acidosis (including ketoacidosis) in 11 patients (21.56%) and sepsis in 10 patients (19.60%) (Table 4).

**TABLE 4:** Concomitant medication (co-suspect, interacting, and concomitant) and associated conditions in fatal ARF reports.

Concomitant medication	N
Biguanide	22
Insulin	14
DPP-4 inhibitors	11
*Diuretics	11
*ACE inhibitors	5
GLP-1 receptor agonist	4
*ARB	6
Sulfonylurea	9
*NSAIDS	4
Statin	10
Acetylsalicylic acid	5
**Associated diseases**	
Type 2 diabetes mellitus	22
Hypertension	5
Pain	4
Blood cholesterol increased	1
Depression	1
Cardiac failure	4
Thyroid disorder	3
Gastroesophageal reflux disease	1
**Associated PTs**	N
Diabetic ketoacidosis, ketoacidosis, and euglycemic diabetic ketoacidosis	15
Dehydration, vomiting, and diarrhea	13
Sepsis, septic shock, and urosepsis	12
Osteomyelitis and acute osteomyelitis	1
Metabolic acidosis and lactic acidosis	10
Urinary tract infection	8
Leg/foot amputation	2
Gangrene	1
Hyperkalemia	2
Myocardial infarction	6
Encephalopathy and metabolic encephalopathy	4
Fournier’s gangrene	1
Hypotension	3
Pancreatitis	3
Cerebrovascular accident	2
Cardiogenic shock	2

ARF, acute renal failure; ICSR, individual case safety report; N, total number of cases; n, number of cases in a given category; DPP-4, dipeptidyl peptidase-4; GLP-1, glucagon-like peptide-1; ACE, angiotensin-converting enzyme; ARB, angiotensin receptor blocker; NSAIDS, nonsteroidal antiinflammatory drugs; PT, preferred term; *, drugs that may induce acute kidney injury (AKI) (Perazella and Rosner, 2022b); drug indication was not mentioned in all reports;one ICSR may include more than one ARF PT reported, and one ICSR may have as suspect or interacting drugs more than one gliflozins, and this may lead to a discrepancy between the number of cases and number of drugs.

1 Introductory Guide for Standardised MedDRA Queries (SMQs) Version 24.1 ACKNOWLEDGEMENTS (2021)

### 3.4 Disproportionality Analysis

#### 3.4.1 ARF

A PRR of *4.68* (95% CI 4.53–4.83) for the association ARF narrow SMQ–gliflozins (all) as compared to ARF–other drugs was found. When we used all database as reference, we found 28 significant associations between different gliflozins use and acute kidney injury, renal failure, renal impairment, dialysis, anuria, and prerenal failure PTs (Figure 2).

In the sensitivity analysis, the significance (as defined by PRR≥1, associated with≥5 cases) of these association was kept for most of the pairs 27) when taking into account only ARF cases where gliflozins were the single suspected drug. When reference was set as all reactions reported to antidiabetics instead of the entire database, 20 of the aforementioned associations were significant in terms of PRR. Narrowing further the analysis to gliflozins as single suspected drugs versus the reports to antidiabetics, we found significant PRRs in 18 pairs (Figure 2).

#### 3.4.2 Nephrolithiasis

**FIGURE 3:**
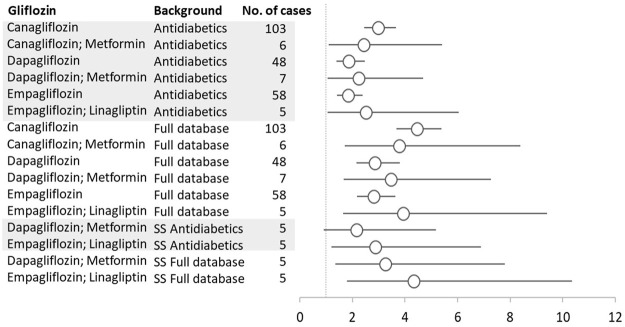
Gliflozins–Nephrolithiasis reactions with the proportional reporting ratio (PRR) greater than 1 and minimum five reactions were reported. SS, single suspect; circles represent PRR, and vertical lines represent 95% confidence.

For nephrolithiasis, we found a PRR of 3.44 (95% CI 3.00–3.95) for all gliflozins calculated versus the entire database. When we took into account all reports where gliflozins were deemed suspected/interacting drugs, we found six pairs of gliflozins–nephrolithiasis with significant PRRs (against both the entire database and reports to antidiabetics). When narrowing to reports where gliflozins were the only suspected drug, we found significant PRRs only for dapagliflozin + metformin and empagliflozin + linagliptin combinations (Figure 3).

## 4 Discussion

Renal safety of SGLTi is much debated in the medical literature. While the benefits of gliflozins in the chronic mild or moderate kidney disease are widely recognized, they are also suspected of inducing adverse renal effects, like acute kidney injury and nephrolithiasis.

Our research focused on the association of gliflozins and ARF and nephrolithiasis in the world’s largest pharmacovigilance database (VigiBase), with the aim to characterize the patients, reports, and drugs involved and also to look into the disproportionality analysis.

Most of both the ARF and nephrolithiasis reactions were reported in patients within the 45–64 years of age group and slightly more in males. This is in line with results of Perlman et al. (2017) who reported a mean age of 59 years and 49% females in their FDA adverse event report system (FAERS) database for the ARF broad SMQ.

Some articles reported that SGLT2 inhibitors were not observed to be associated with increased risk for AKI. The JADER database study (Katsuhara and Ogawa, 2020) did not find a significant association between SGLT2 inhibitors and ARF, but 1) the database is smaller (only 126 ARF cases reported to SGLT2) and also 2) they used ARF broad definition and included 50 PTs, which may have affected their ROR. One network meta-analysis (Zhao et al., 2020) only included clinical trials on patients with established or at risk of cardiovascular disease or CKD, limiting its generalizability to other patient populations without these risks, and since the renal risk was not the primary objective, the AKI events were more likely to be underreported. Also, the definitions of ARF may differ between the trials included. On the other hand, case reports of ARF due to SGLT2i with proper documentation are published, and ADR reports continue to be submitted to VigiBase, despite the FDA strengthening the warning on the risk of AKI in October 2015, so its effect should be faded.

The FDA warning on the risk of AKI associated with gliflozins was issued in June 2016 and probably impacted the reporting of ARF in the VigiBase. The number of ARF reports doubled from 2015 to 2017 (341–720), but the highest number of ARF cases was in 2018 (1186 reports). Perlman et al. (2017) found a rise of 1 in ROR after the FDA warning and by September 2016 (a period of approximately 2 months. Interestingly, for ARF, a lot of the reports came from the consumers only (59.57% of the notifiers with available information), although typically this is not a reaction that can be recognized by consumers. We could not identify the reason for this reporting and how much this impacted the disproportionality analysis. A dose–response relationship could not be established, as variation in the number of cases *per* dose was probably due to the patterns of the gliflozins’ use (e.g., approximately the same number of cases for canagliflozin 100 and 300 mg, more case for dapagliflozin 10 versus 5 mg, but also more cases for empagliflozin 10 versus 25 mg). This is consistent with the dose trend of reports from another analysis that looked at the gliflozins reactions in VigiBase during the same period (Frent et al., 2021).

The exact mechanism for gliflozins induced AKI is not fully understood. Decreased glomerular pressure is a likely potential explanation, but development of osmotic nephropathy may be also an alternative explanation at least in some reports. Some case reports with biopsy evidence of osmotic nephropathy were published (Sia et al., 2021; Perazella and Juncos, 2022). A possible explanation is that proximal tubules are exposed to significant amounts of filtered glucose, as there are some experimental studies and in humans exposed to glucose 10% solutions that leads to osmotic nephropathy. Probably, the excessive amount of urinary glucose undergoes tubular cell pinocytosis. Most of the AKI cases occurring in clinical practice were not biopsied; therefore, drug-induced osmotic nephropathy as a possible mechanism for AKI induced by SGLT2i is probably under-recognized (Perazella and Juncos, 2022). A possible approach mentioned in the literature is to undertake a kidney biopsy in patients with prolonged AKI (>5 days) or dialysis-dependent AKI that persists despite withdrawing SGLT2i, as such a clinical presentation is unlikely only due to a reduction in glomerular pressure or water and sodium depletion (Phadke et al., 2020). In our analysis, in 92.43%% of patients with ARF where gliflozin was discontinued, the reaction abated and most patients improved after stopping the drug. This is in line with the strengthening warning of the FDA (U.S. Food and Drug Administration, 2016).

The FDA warning was based on the analysis of 101 cases of AKI reported during March 2013–October 2015 with approximately half of the AKI occurring within 1 month of starting the drug (U.S. Food and Drug Administration, 2016). In our analysis, only 36.32% of the cases with available information occurred within 1 month and another 14.59% occurred during 1–2 months of starting therapy. It is well known that the initiation of SGLT2i may cause an increase in serum creatinine and decrease in eGFR during the first 6 weeks of therapy. In patients with moderate renal impairment, the increase in serum creatinine generally does not exceed 0.2 mg/dl, occurs within the first 6 weeks of starting therapy, and then stabilizes. Increases that do not fit this pattern should prompt further evaluation to exclude the possibility of acute kidney injury (AstraZeneca Pharmaceuticals, 2021; Janssen Pharmaceuticals, 2021; Boehringer Ingelheim Pharmaceuticals, 2022). However, in VigiBase almost half of the cases (49%) with available data occurred after 2 months of SGLT2i therapy. A descriptive study of clinical spectrum and mechanism of AKI in patients with diabetes mellitus on SGLT2i found a wide interval of 7–365 days period after initiation of SGLT2i for AKI (Pearlman et al., 2018).

There are some known risk factors that may predispose patients with SGLT2i treatment to AKI, like decreased blood volume, chronic kidney insufficiency, congestive heart failure, and concomitant medications such as diuretics, ACE inhibitors, ARBs, and NSAIDs (U.S. Food and Drug Administration, 2016).

Since the cause of AKI due to gliflozins is generally attributed to osmotic diuresis that induce plasmatic volume depletion (AstraZeneca Pharmaceuticals, 2021; Janssen Pharmaceuticals, 2021; Boehringer Ingelheim Pharmaceuticals, 2022), we looked at the associated conditions of the patients like dehydration, diarrhea and vomiting, cardiac failure, hypovolemia, or concomitant use of diuretics. Regarding other medication that can cause/contribute to ARF, we found a significant number of patients with diuretics, ACE inhibitors, ARB blockers, and NSAIDs. This is still far from the findings of Pearlman et al. (2018), where all patients with gliflozins-associated AKI had been prescribed RAAS blockers and most had also injection of contrast-product or NSAIDs).

The most associated reaction in ARF cases was ketoacidosis in 27.87% of patients. Diabetic ketoacidosis (DKA) is a known rare but potentially life-threatening condition also related to SGLT2i (AstraZeneca Pharmaceuticals, 2021; Janssen Pharmaceuticals, 2021; Boehringer Ingelheim Pharmaceuticals, 2022); Plewa et al., 2022). Due to glucose-induced osmotic polyuria and even emesis, volume depletion is a major cause of AKI in DKA patients. AKI is a complication of diabetic ketoacidosis and an independent risk factor for poor long-term renal outcomes and mortality in DKA patients (Orban et al., 2014; Chen et al., 2020; Janssen Pharmaceuticals, 2021).

Sepsis and septic shock were the second most common reactions reported together with ARF in our analysis (n = 366 reactions reported). Sepsis is a known complication of diabetes mellitus and a major cause of AKI. Diabetes was also identified as an independent risk factor for AKI. Moreover, the risk of AKI during sepsis is increased in patients with diabetes. AKI develops in one-fourth of patients with sepsis and half of the patients with shock and is associated with high-mortality (up to 70%) (Venot et al., 2015) ARF with fatal outcome.

In our study, the reported data for time-to-onset was limited, only 10 cases out of 54 cases of fatal ARF, with 5 cases reported within 6 weeks of treatment with gliflozin. Fatal ARF may occur also within 6 weeks of the treatment with gliflozin, when an acute reversible decrease in eGFR is expected. AKI that may lead to hospitalization or dialysis was previously reported in SGLT2i US Prescribing Information (US PI), but AKI with fatal outcome was not considered as expected with gliflozins (fatalities were not mentioned in the FDA strengthened warning or in the SGLT2i US PI) (U.S. Food and Drug Administration, 2016; Janssen Pharmaceuticals, 2021; AstraZeneca Pharmaceuticals, 2021; Boehringer Ingelheim Pharmaceuticals, 2022). Medical literature presents though scattered reports suggesting that the risk for AKI may occasionally be fatal or might require renal replacement therapy (Szalat et al., 2018) and we provide in our analysis additional evidence showing that fatal ARF associated with the use of gliflozins is possible, and even more, this may also occur within first 6 weeks of gliflozin treatment.

### 4.1 Nephrolithiasis

SGLT2i treatment could lower the risk for incident and recurrent kidney stones in people with type 2 diabetes, as recent evidence suggests (Kristensen et al., 2021; Balasubramanian et al., 2022). However, it was also suggested that SGLT2i can cause hyperuricosuria (Chino et al., 2014; Novikov et al., 2019), which may confer a greater risk for specific types of kidney stones. Moreover, a recent study of pooled analysis of data from clinical studies found that in 19% of patients, treatment with luseogliflozin resulted in increased serum uric acid, which may be due to reduced glomerular filtration of uric acid via the tubuloglomerular feedback (Chino et al., 2022). Yet, another meta-analysis found no association of SGLT-2 inhibitors with nephrolithiasis (Cosentino et al., 2019).

In 2017, a potential signal of nephrolithiasis in patients using SGLT2 inhibitors was detected by the FDA, but the decision was not to update the product information based on available information at that time (U.S. Food and Drug Administration, 2017). Our analysis does not contradict previous studies with conflicting results; it only adds the global perspective from VigiBase, the largest database of spontaneous reported ADRs and we included data until September 2021; therefore, data reported from four additional years were included. In fact, the highest evidence till present is the one the meta-analyses of randomized controlled trials like, where no association has been observed (Cosentino et al., 2019). In VigiBase, fewer nephrolithiasis reactions (231) were reported than ARF reactions (3,972), and these did not seem to have been impacted by the FDA potential signal from beginning of 2017. Positive de-challenge was noticed in 20% from the total of 231 reactions, but no cases with positive re-challenge were reported. The most frequently associated adverse event with nephrolithiasis was urinary/renal infection (52 reports, 22.51%). Kidney stone formers are at increased risk of developing urinary tract infections. Some potential mechanisms have been postulated, like bacteria, which increase aggregation of crystals or increase expression of a stone matrix (Brain et al., 2021).

Although we found disproportionality for gliflozins–nephrolithiasis reactions, the low number of reactions reported and the fact that most of them come from the USA, where all adverse events are reported, whether or not implying a causality relationship, firm conclusions regarding SGLT2i-induced nephrolithiasis are still debatable.

### 4.2 Disproportionality Analysis

The disproportionality analysis showed an increased risk of ARF and gliflozins (PRR = 4.67). From the total of 28 gliflozin-ARF PTs associations found significant in the general analysis, 20 kept their significance when the background was restricted to antidiabetics’ events (considering only reactions to antidiabetics as background). This analysis was done in order to take into account the increased baseline risk of ARF in diabetes patients. In the scope of eliminating alternative causes’ risk, we calculated the PRRs for reactions where only gliflozins were suspected and we found that almost all pairs from the general analysis kept their significance. We can therefore say that our findings for the ARF SMQ narrow are consistent with the ones from the 2016 study (Perlman et al., 2017). Likewise, for nephrolithiasis, our case–non case analysis showed a significant disproportionality (PRR = 3.44) in the general analysis and the same pairs kept significance with antidiabetics as the background. However, it is important to notice that only fixed combinations of gliflozins and metformin/linagliptin kept their significant PRR when excluding nephrolithiasis reports with other suspected drugs. More research is needed to investigate these associations.

Our analysis adds to the current knowledge the description of the gliflozins-related ARF cases from VigiBase and the description of fatal cases. We would like to emphasize here the judicious usage of gliflozins in patients with factors that may predispose patients to ARF. Treating physicians should be aware that AKI is a serious adverse event with potential fatalities when gliflozin class is used. Fatal ARF may occur also within 6 weeks of the treatment with gliflozin, when an acute reversible decrease in eGFR is expected; however, our observation is based on limited time-to-onset data. A high prevalence of severe infections (sepsis) was found in the patients with ARF, and this finding strengthens the link between sepsis and ARF.

### 4.3 Limitations

1. Another study on ARF reports related to gliflozins was published and contained data from the FDA adverse event report system, FAERS database, until September 2016 (Perlman et al., 2017). We are adding a global perspective, as the information is from VigiBase, the largest database of spontaneous reported ADRs and for an extended reporting period, until 31 August 2021.
2. Most of the ARF reports came from spontaneous reporting, the majority from consumers followed by healthcare professionals (60.86% and 38.09% from available data, respectively with 59.57% coming from consumers only). Therefore, almost 60% of the cases were not medically confirmed. Meanwhile, half of the fatal ARF cases were considered medically confirmed.
3. The disproportionality method: The real risk of ADRs cannot be measured, but only the difference in the rate of notifications by calculating the PRR. However, this method is useful for detecting health risks that need further investigation.
4. Underreporting, lack of reported clinical details, including laboratory results, and reporting bias (more likely to report severe cases).
5. The information contained in the VigiBase comes from a variety of sources, the amount of information in each report varies between reports, and the probability that the suspected adverse effect is drug-related is not the same in all cases. Variation in the causality relation between the drug and the ADR may have affected our analysis since approximately 80% of ARF reports and 84% of nephrolithiasis reports come from the regions of America of which the United States of America (United States) may have a great contribution. The United States collects any adverse event associated with the use of a drug in humans, whether or not considered drug-related (eCFR—Code of Federal Regulations, 2020), while other countries collect only suspected ADRs with at least a possibility of a causal relationship between the drug and reported (Vigiaccess, 2022) Also, the frequency of reporting to VigiBase may vary over time and between countries. This could be the case for increased reporting of AKI following the FDA warning (U.S. Food and Drug Administration, 2016), which could have resulted in increased recognition and reporting of AKI (notoriety bias).

## 5 Conclusion

SGLT2i are considered safe and provide multiple benefits for patients with type 2 diabetes mellitus. Exposure of patients to SGLT2i is expected to increase due to the recent evidence of cardiovascular and renal protection that led to extension of their indications. ARF cases are rarely reported and the benefit–risk balance of these antidiabetics remains favorable. Nevertheless, healthcare professionals and patients should be aware that: 1) AKI may occur following the use of SGLT2i and may produce serious and even life-threatening consequences, 2) especially in the context of concomitant diseases or medications affecting the renal mechanisms, and 3) also fatal ARF may occur within the first 6 weeks (however, our observation is based on limited time-to-onset data) of treatment with gliflozin, when an acute reversible decrease in eGFR is expected. The observational nature of this study precludes firm statements, but the importance of the findings demands future in-depth analyses to explore the relation with the fatal outcome.

## Data Availability

The data that support the findings of this study are available from the WHO Uppsala Monitoring Center, but restrictions apply to the availability of these data, which were used under license for the current study, and so are not publicly available. Requests to access these datasets should be directed to: CustomSearches@who-umc.org.
