# Supplementary material for: A Description of Acute Renal Failure and Nephrolithiasis Associated With Sodium–Glucose Co-Transporter 2 Inhibitor Use: A VigiBase Study
Source: Front Pharmacol. 2022 Aug 8;13:925805. doi: 10.3389/fphar.2022.925805 (PMC9393368; doi:10.3389/fphar.2022.925805)
Supplement: Supplementary file 1 [file Table1.DOCX]

**Article title: A description of acute renal failure and nephrolithiasis associated with sodium glucose co-transporter 2 inhibitors use: A VigiBase study**

**Journal: Frontiers in Pharmacology**

**Authors: Ioana Frent, Daniel Leucuta, Camelia Bucsa, Andreea Farcas, Florin Casoinic Cristina Mogosan, correspondence to Daniel Leucuta, at** [**dleucuta@umfcluj.ro**](mailto:dleucuta@umfcluj.ro)

**Supplementary Table 1**: Calculation of the proportional reporting ratio (PRR) for Acute renal failure (ARF)

|  | Number of cases of ADRs with ARF | Number of other ADRs | Total |
| --- | --- | --- | --- |
| Studied drug | a | b | a + b |
| Other drugs | c | d | c + d |

**PRR=[a/(a+b)]/c/(c+d)**

**PRR=4.67**

ADR: adverse drug reaction; ARF: Acute renal failure (Preferred terms from ARF SMQ Narrow were taken up for consideration)

a: the number of exposed cases (cases of ARF with the studied drugs; fixed drug combinations of gliflozins were excluded); b: the number of exposed non-cases (all ADRs other than ARF with the studied drug); c: the number of non-exposed cases (ARF with other drugs); d: the number of unexposed non-cases (all adverse drug reactions other than ARF with other drugs).

Total number of adverse drug reactions reported in VigiBase® up to 31 Aug 2021: a + b + c + d

a=3.702

b=58.527

c= 347.140

d=26.961.044

**Supplementary Table 2**: Calculation of the proportional reporting ratio (PRR) for Nephrolithiasis

|  | Number of cases of ADRs with Nephrolithiasis | Number of other ADRs | Total |
| --- | --- | --- | --- |
| Studied drug | a | b | a + b |
| Other drugs | c | d | c + d |

**PRR=[a/(a+b)]/c/(c+d)**

**PRR=3.44**

ADR: adverse drug reaction;

a: the number of exposed cases (cases of Nephrolithiasis with the studied drugs; fixed drug combinations were excluded); b: the number of exposed non-cases (all ADRs other than Nephrolithiasis with the studied drug); c: the number of non-exposed cases (Nephrolithiasis with other drugs); d: the number of unexposed non-cases (all adverse drug reactions other than Nephrolithiasis with other drugs).

Total number of adverse drug reactions reported in VigiBase® up to 31 Aug 2021: a + b + c + d

a=210

b=59.563

c=27.851

d=27.282.789
